# Loss of Cullin 5 in myeloid cells protects against autoimmune neuroinflammation

**DOI:** 10.3389/fimmu.2025.1611818

**Published:** 2025-08-06

**Authors:** Yohaniz Ortega-Burgos, Asif A. Dar, Siera A. Tomishima, Ipsita Guha, Carleigh O’ Brien, Nadia Porter, F. Chris Bennett, Paula M. Oliver

**Affiliations:** ^1^ Department of Pathology and Laboratory Medicine, Perelman School of Medicine, University of Pennsylvania, Philadelphia, PA, United States; ^2^ Division of Protective Immunity, Children’s Hospital of Philadelphia, Philadelphia, PA, United States; ^3^ Department of Neurology, Perelman School of Medicine, University of Pennsylvania, Philadelphia, PA, United States; ^4^ Department of Psychiatry, Perelman School of Medicine, University of Pennsylvania, Philadelphia, PA, United States; ^5^ Division of Neurology, Department of Pediatrics, Children’s Hospital of Philadelphia, Philadelphia, PA, United States

**Keywords:** Cullin 5, neuroinflammation, macrophages, ubiquitin ligase (E3), macrophage polarization

## Abstract

Autoimmune neuroinflammation occurs when an individual’s immune cells attack the brain, spinal cord or peripheral nerves. Several Suppressor of Cytokine Signaling (SOCS) proteins have been shown to limit pro-inflammatory signaling pathways in myeloid cells and prevent neuroinflammation. They rely on several mechanisms to accomplish this. Their SH2 domain allows them to bind phosphorylated tyrosine residues on surface receptors to prevent downstream signaling while their C-terminal SOCS domain can promote their assembly with Cullin5 (CUL5) to degrade signaling proteins. To date, the role of CUL5 in myeloid-cell-mediated function is poorly understood. Here we show that loss of *Cul5* in myeloid cells resulted in reduced neuroinflammation and attenuated progression of Experimental Autoimmune Encephalomyelitis (EAE). Although peripheral CD4^+^ T cell activation was not overtly affected, *Cul5*-deficient macrophages in the Central Nervous System (CNS) demonstrated a significant shift toward an anti-inflammatory phenotype, characterized by increased expression of Arginase 1. This correlated with an enhanced frequency of FoxP3^+^ regulatory T cells. In contrast to what would be predicted if CUL5 and SOCS proteins work together to degrade pro-inflammatory cytokine signaling, *Cul5* deletion in myeloid cells selectively enhanced IL-4-mediated Arginase 1 expression. These findings identify CUL5 as an unanticipated pro-inflammatory mediator during neuroinflammation and reveal its potential as a therapeutic target for autoimmune diseases.

## Introduction

Macrophages are essential components of both the innate and adaptive immune systems, often considered a bridge between the two (1). The majority of these cells emerge from hematopoietic stem cells (HSCs) in the bone marrow (2). They play critical roles in tissue repair, phagocytosis, antigen presentation, immune surveillance, and inflammation (3, 4). While macrophages have an extensive repertoire of phenotypic characteristics, they are often broadly categorized into pro-inflammatory and anti-inflammatory (5–7). These functions allow them to mediate inflammation and resolution in autoimmune diseases, such as Multiple Sclerosis (MS) (8–11).

Multiple Sclerosis is a chronic, inflammatory and neurodegenerative disease of the Central Nervous System, characterized by the immune-mediated recognition of myelin or other neuronal antigens, triggering demyelination (12–14). This can result in motor and sensory dysfunctions, cognitive impairment, and disability (15). Over the past decade, the number of people with MS has increased worldwide (16) and although its pathology involves a complex interplay between autoreactive T cells, B cells and other immune subsets, macrophages are pivotal in mediating CNS damage.

In MS and its mouse model, EAE, macrophages release a variety of pro-inflammatory cytokines, including TNF-α, IL-1β, and IL-6, which contribute to the recruitment of additional immune cells and increase blood-brain barrier (BBB) permeability (17, 18). These cytokines exacerbate neuroinflammation and promote the damage of oligodendrocytes and myelin (19). However, during the later phase of inflammation, macrophages secrete several anti-inflammatory cytokines such as IL-10 and TGF-β, which are needed to support tissue repair and remyelination (20, 21). The balance between pro-inflammatory and anti-inflammatory cytokine signaling in macrophages determines disease progression and outcomes in MS. (22). While many regulatory factors remain unknown, the SOCS family is one of the key groups of the proteins that control macrophage phenotypes.

There are eight SOCS proteins, SOCS1–7 and CIS (23), that employ diverse mechanisms to regulate cytokine signaling in macrophages (24–26). SOCS proteins can directly inhibit kinase activity via their KIR domain (26), block signaling via their SH2 domain (27, 28), or promote ubiquitination of signaling intermediates via their SOCS domain (24–26). Specifically, the SOCS domain binds the E3 ubiquitin ligase CUL5 (29). However, SOCS proteins bind CUL5 with varying affinities (30). Thus, the extent to which the SOCS proteins work with CUL5 to ubiquitinate substrates remains unclear.

SOCS1 and SOCS3 are the most well-characterized SOCS-family members regarding macrophage function and neuroinflammation. Both SOCS1 and SOCS3 limit pro-inflammatory macrophage polarization using distinct mechanisms (31, 32). Furthermore, both SOCS1 and SOCS3 protect mice from developing EAE. Specifically, myeloid deficiency of SOCS3 results in an atypical and progressive form of EAE, mediated by enhanced neutrophil accumulation and ROS production (33). Consistent with the role of SOCS1 in macrophage activation of T cells, treating mice with a SOCS1 KIR reduced IL-17 production by T cells and prevented the development of EAE (34). Whether SOCS proteins limit neuroinflammation by working with CUL5, and the role of CUL5 in macrophage function and neuroinflammation remains unclear.

Here, we investigated how myeloid deletion of *Cul5* impacted macrophage function and EAE progression. In contrast to what was observed in SOCS3-deficient mice, we found that mice lacking *Cul5* in myeloid cells (*Cul5^fl/fl^* LysM-Cre mice) were protected against progression of neuroinflammation following EAE induction. *Cul5^fl/fl^* LysM-Cre mice showed a significant decrease in the numbers of multiple immune cell subsets, including CD4^+^ T cells, in the CNS at the peak of disease. Decreased EAE progression was not due to a reduction of T cell activation in secondary lymphoid organs nor due to a reduced number of T cells infiltrating the CNS. Rather, we found a significant increase in the frequency of Arginase 1+ macrophages in the CNS of *Cul5^fl/fl^* LysM-Cre mice. Supporting this, *Cul5*-deficient bone marrow-derived macrophages (BMDMs) were more likely to increase Arginase 1 expression when cultured with IL-4. Furthermore, *Cul5*-deficient macrophages isolated from the CNS during EAE progression showed increased levels of multiple proteins associated with the suppression of inflammation. This study uncovers a key role for CUL5 in regulating macrophage function and in modulating the progression of neuroinflammation, highlighting its significance as a regulatory factor in immune-driven CNS pathology.

## Materials and methods

### Mice

*Cul5^fl/fl^* LysM-Cre mice were generated by crossing *Cul5^fl/fl^* mice with LysM^Cre^ mice (Jackson Laboratory, Bar Harbor, ME, strain #004781) to obtain LysM^Cre^ *Cul5^fl/fl^* mice. Male and female mice with conditional deletion of Cul5 are referred to as *Cul5^fl/fl^* LysM-Cre and these mice are on a C57BL/6 background. To the extent possible, LysM-Cre littermates were used as controls. All mice were bred in-house under specific pathogen-free conditions in the animal facility at the Children’s Hospital of Philadelphia (CHOP). The mice were housed at 18–23°C with 40–60% humidity, with 12-h light/12-h dark cycles. All mice, unless stated otherwise, were 8–12 weeks of age, and both sexes were used without randomization or blinding. Animal housing, care, and experimental procedures were performed in compliance with the CHOP Institutional Animal Care and Use Committee.

### EAE induction

*Cul5^fl/fl^* LysM-Cre and WT mice were injected s.c. with 200 μg of MOG_35–55_ (Genemed Synthesis) emulsified in 5 mg/ml CFA (BD Biosciences). On the same day and again 48 hours later, mice received an intraperitoneal injection of 200 ng of pertussis toxin (Sigma-Aldrich). As negative controls, mice were left unimmunized. Mice were then examined daily for the clinical signs of EAE in a blinded fashion. The disease severity of EAE was scored using the standard clinical score as previously described: 0, no disease; 0.5, distal limp tail; 1, compete limp tail; 1.5, limp tail and hindlimb weakness; 2, unilateral partial hindlimb paralysis; 2.5, bilateral partial hindlimb paralysis; 3, complete bilateral hindlimb paralysis; 3.5 complete bilateral hindlimb paralysis and partial forelimb paralysis; 4, moribund (completely paralyzed); 5, death.

### CNS isolation and preparation of single-cell suspensions

Mice were euthanized according to IACUC guidelines and perfused with PBS through the left ventricle of the heart using a 25G butterfly needle attached to a 10-mL syringe. Spinal cord, brain, and brain stem were removed by dissection. CNS tissues was processed through mechanical dissociation in 5 ml of digestion medium (collagenase I and Ia (Sigma-Aldrich, catalog nos. C0130 and C9891, respectively), and 20 μg/ml DNAse I (Roche, catalog no. 10104159001) and digested at 37°C for 30 min. Enzymatic digestion was stopped by adding EDTA to a final concentration of 5 mM. To homogenize the tissue, the suspension was repeatedly sucked up and ejected using a 5-ml syringe with an 18G needle until a uniform milky homogenate was formed, avoiding excessive foaming. The homogenate was filtered through a 70-μm nylon cell strainer into a 50-ml Falcon tube, which was then filled with media (RPMI 1640 with 1% FCS) and centrifuged at 1300 rpm for 8 min at 4°C to pellet the cells and myelin. The supernatant was discarded by pouring it off gently, and the pellet was resuspended in a final volume of 5 ml of 30% Percoll and mixed. The 30% Percoll-containing CNS mixture was poured gently into a 15-ml tube containing 5 ml of 70% Percoll and then centrifuged at 1800 rpm for 30 min at 4°C, without brakes. After centrifugation, a PBS–Percoll gradient formed, with each layer containing a specific fraction of the homogenate. The myelin was carefully sucked off using a suction pump or pipette. The large middle layer containing the leukocytes was transferred without the bottom layer of RBCs into a Falcon tube. The supernatant was discarded, and the cells were resuspended in media (RPMI 1640 with 1% FCS).

### Bone marrow derived macrophages

Bone marrow-derived macrophages were generated from the femurs and tibias of mice as previously described (35). Mice were euthanized according to institutional animal care guidelines, and femurs and tibias were harvested under sterile conditions. Bone marrow cells were flushed from the bones using a 25G needle and DMEM+GlutaMAX ^™^(Gibco), medium supplemented with 10% fetal bovine serum (FBS) and 1% penicillin-streptomycin. The cell suspension was filtered through a 70 µm cell strainer to remove debris and centrifuged at 400 × g for 7 minutes at room temperature. The pellet was resuspended in red blood cell lysis buffer for 2 minutes at room temperature, washed with PBS, and plated in non-tissue culture-treated Petri dishes at a density of 5 × 10^6^ cells/mL in differentiation media.

Differentiation media consisted of complete DMEM+GlutaMAX ^™^ supplemented with 10 ng/mL macrophage colony-stimulating factor (M-CSF; PeproTech). Cells were incubated at 37°C, 5% CO_2_, with fresh media containing 10 ng/mL M-CSF replenished on day 3. By day 6, adherent cells were considered fully differentiated BMDMs. Macrophage identity was confirmed by flow cytometry for CD11b and F4/80 expression and morphological assessment under a light microscope.

For experimental assays, BMDMs were detached using ice-cold PBS containing 2 mM EDTA and collected for downstream applications. Cells were either stimulated with IL-4 (PeproTech) 10ng/mL, IL-13 10ng/mL (PeproTech), LPS 100ng/mL (Sigma Aldrich) or IFN-γ (PeproTech) 10ng/mL for the time points indicated.

### Flow cytometry

Single-cell suspensions were stained with a fixable viability dye (LIVE/DEAD fixable blue stain), then pretreated with unlabeled anti-CD16/CD32 (Fc Block, BD Pharmingen). Cells were then stained in FACS buffer (PBS containing 2.5% FCS and 0.1% sodium azide) with mixtures of directly conjugated Abs.

For intracellular cytokine staining, a single-cell suspension was incubated with MOG_35–55_ (Ge (30 ng/ml; Genemed Synthesis) and ionomycin (1 μM; Abcam) in the presence of GolgiPlug (1 μg/ml; BD Biosciences, catalog no. 555029) and GolgiStop (1 μg/ml; BD Biosciences, catalog no. 554724) for 4 h at 37°C and stained using the Cytofix/Cytoperm kit (BD Biosciences). For Foxp3 staining, a Foxp3 staining kit (eBioscience) was used according to the manufacturer’s instruction. Samples were analyzed using an LSRFortessa flow cytometer (BD Biosciences) at the CHOP Flow Cytometry Core Facility. Data were analyzed using FlowJo software v10 (Tree Star, Ashland, OR). Results are expressed as the percentage of positive cells or median fluorescence intensity (MFI).

### Western blotting

Bone marrow-derived macrophages (BMDMs) were generated as described previously and collected on day 6 post-differentiation. Cells were washed twice with ice-cold PBS and lysed in RIPA buffer (50 mM Tris-HCl pH 7.4, 150 mM NaCl, 1% NP-40, 0.5% sodium deoxycholate, 0.1% SDS) supplemented with protease and phosphatase inhibitors. Lysates were incubated on ice for 30 minutes, followed by centrifugation at 14,000 × g for 10 minutes at 4°C. Supernatants were collected, and protein concentrations were determined using the BCA protein assay kit.

SDS-PAGE and protein transfer: Equal amounts of protein (20–40 µg) were mixed with 2× Laemmli sample buffer containing β-mercaptoethanol and boiled at 95°C for 5 minutes. Samples were loaded onto a 4–12% Bis-Tris polyacrylamide gel and separated by SDS-PAGE at 120V for ~120 minutes in 1× running buffer. Proteins were transferred onto a membrane using a wet transfer system at 40V for 120 minutes.

Immunoblotting: Membranes were blocked with TBS-Tween (TBS-T, 0.1% Tween-20) for 1 hour at room temperature. Membranes were incubated overnight at 4°C with primary antibodies diluted in blocking buffer. After washing three times with TBS-T (5 minutes each), membranes were incubated with secondary antibodies (1:5,000) for 1 hour at room temperature.

### Whole-cell proteomics data acquisition and processing

*Cul5^fl/fl^* LysM-Cre and WT mice were induced with EAE (as previously described). CNS was collected at day 12 post-induction and cells was sorted for CD45^hi^ CD11b^+^ macrophages. Data were processed using R for statistical analysis. Results were quantile-normalized and analyzed for differential abundance. Differentially abundant proteins were identified using a Limma t-test, and protein identifiers were mapped to their corresponding gene names. Gene-level data were subsequently analyzed for enrichment using the MSigDB Hallmark Gene Dataset. The heatmap was generated using the heatmap package, displaying the top 20 upregulated and downregulated genes. The volcano plot was generated with a significance threshold of *p* < 0.05.

### In-solution digestion

Sorted cells underwent lysis and solubilization in 2% SDS-based lysis buffer. Lysate was reduced and alkylated before undergoing protein aggregation capture (PAC) cleanup and in-solution digestion with Trypsin/LysC in an automated KingFisher APEX system (68). The resulting peptides were desalted using C18 stage tips, dried via vacuum centrifugation, and reconstituted in 0.1% TFA containing iRT peptides.

### Mass spectrometry acquisition and data analysis

Peptides were analyzed on a TimsTOF Pro2 mass spectrometer coupled with a NanoElute 2 nanoLC system using data independent acquisition (DIA). Subsequently, raw data was searched using Spectronaut (69), and bioinformatics analysis was conducted in R.

## Results

### Myeloid restricted *Cul5* deletion does not significantly impact lineage development

**Figure 1:**
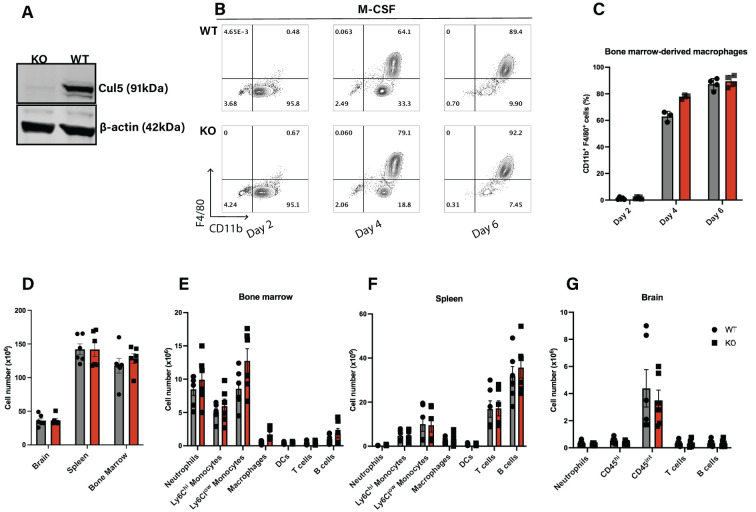
Cul5 does not broadly affect the differentiation of myeloid cells under homeostatic conditions. **(A)** Western blot analysis of CUL5 expression in BMDMs from *Cul5fl/fl* LysM-Cre and WT mice. β-actin is shown as a loading control. **(B)** Representative flow cytometry plots showing WT and *Cul5*-deficient BMDM differentiation at days 2, 4, and 6 in culture, (n=4 per group). **(C)** Compiled data from bone marrow-derived macrophage differentiation from *Cul5fl/fl* LysM-Cre and WT mice. Bars indicate mean ± SEM from two independent experiments. **(D)** Absolute cell counts in the spleen, bone marrow, and brain of *Cul5fl/fl* LysM-Cre and WT mice under homeostatic conditions. **(E–G)** Flow cytometry analysis of myeloid and lymphoid cell populations in the spleen, bone marrow, and brain. Data were analyzed using multiple unpaired t-tests and are representative of two independent experiments (n=5–6 per group). Each dot represents data from a single mouse.

CUL5 is expressed across various tissues and cell types, playing a crucial role in protein degradation and cellular homeostasis (36). Its expression is particularly enriched in myeloid cells, suggesting a specialized function in regulating immune responses (37). CUL5 protein levels, however, vary depending on cell type and differentiation status (38), and its precise roles in myeloid cell biology, development, differentiation and neuroinflammation remain unexplored. Given that CUL5 works with SOCS-box containing proteins that are known to target signaling mediators (36, 39), particularly those downstream of cytokine receptors, we sought to test whether loss of CUL5- in myeloid cells would impact macrophage development. We generated mice in which *Cul5* is selectively deleted in macrophages, monocytes, and neutrophils by crossing *Cul5^fl/fl^* to LysM-Cre mice to produce *Cul5^fl/fl^* LysM-Cre mice. To confirm that *Cul5* was deleted, we generated BMDMs from WT and *Cul5^fl/fl^* LysM-Cre mice and analyzed CUL5 protein levels by western blot (**Figure 1A**). We found that Cul5 was effectively deleted in BMDMs from *Cul5^fl/fl^* LysM-Cre mice. We next assessed whether loss of *Cul5* impacted macrophage differentiation capacity *in vitro* using bone marrow cells cultured in the presence or M-CSF (**Figures 1B, C**). BMDM cultures from WT and *Cul5^fl/f^* LysM-Cre mice showed similar frequencies of cells expressing F4/80 and CD11b on days 2, 4, and 6 post-seeding (**Figures 1B**), showing no significant differences between the genotypes (**Figure 1C**).

To determine whether loss of *Cul5* impacts myeloid cell differentiation *in vivo*, we assessed WT and *Cul5^fl/fl^* LysM-Cre mice for frequencies and numbers of myeloid and lymphoid subsets using flow cytometry. We focused on organs known to be important for myeloid cell differentiation and sites of neuroinflammation. We found that the overall numbers of cells in these tissues were similar between WT and *Cul5^fl/fl^* LysM-Cre mice (**Figure 1D**). Analysis of specific types of myeloid and lymphoid cells in bone marrow (**Figure 1E**), spleen (**Figure 1F**) and brain (**Figure 1G**) showed no substantial differences in myeloid and lymphoid cell abundance, nor frequency (**Supplementary Figure S1**). These results suggested that the loss of *Cul5* in myeloid cells did not significantly impact the development or differentiation of myeloid lineages under homeostatic conditions.

### Loss of *Cul5* reduces EAE severity

**Figure 2:**
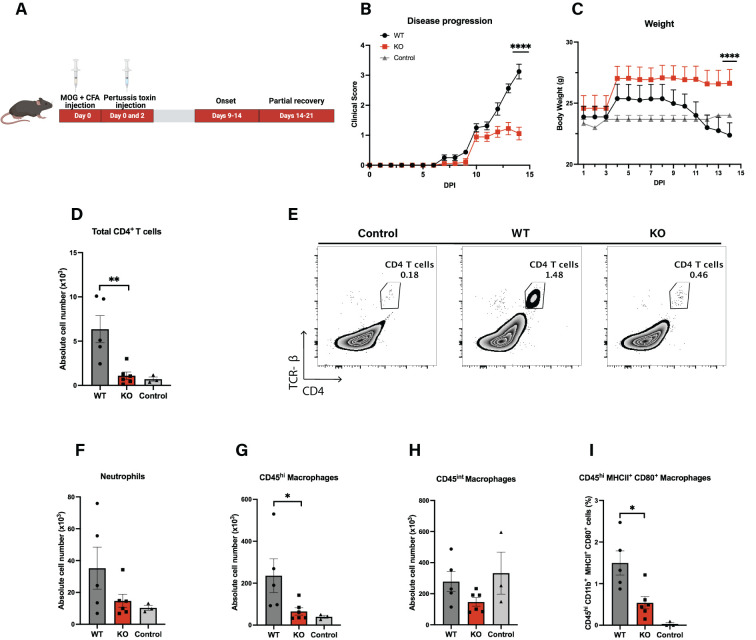
Loss of Cul5 reduces EAE severity. **(A)** Schematic representation of the experimental timeline for EAE induction following *MOG_35–55_* immunization. **(B)** Mean clinical scores of *Cul5^fl/fl^* LysM-Cre and WT mice over the course of the disease. Disease progression was monitored daily until day 14 post-induction (*n* = 5–6 mice per group). **(C)** Mean body weights of MOG immunized mice. (*n* = 5–6 mice per group). Data in **(A–C)** are representative of three independent experiments. **(D)** Representative flow plots of CD4+ T cells in the CNS at day 14 post-EAE induction. **(D–I)** Quantification of immune cell populations infiltrating the CNS at day 14 post-induction, including total CD4^+^ T cells **(E)**, neutrophils **(F)**, CD45^hi^ infiltrating myeloid cells **(G)**, CD45^int^ myeloid cells **(H)**, and MHCII^+^CD80^+^ macrophages **(I)**. Data represent two independent experiments. Error bars indicate mean ± SEM. Statistical significance was determined using Two-way ANOVA **(A, B)** or unpaired t-test **(D–I)** (*P < 0.05, **P < 0.01, ***P < 0.001, ****P < 0.0001).

EAE is the most widely used mouse model of MS recapitulating key pathological features including immune-mediated demyelination and neuroinflammation (40). It is initiated when myelin specific CD4^+^ T cells become activated (40, 41) and the blood-brain barrier (BBB) is disrupted, allowing immune cell infiltration and myelin destruction. While EAE is considered to be T cell-mediated, macrophages play a critical role in disease progression by promoting the reactivation of T cells within the CNS (42). To test the role of Cul5 in myeloid cell-mediated neuroinflammation, EAE was induced in 8-10-week WT and *Cul5^fl/fl^* LysM-Cre mice as illustrated (**Figure 2A**). Mice were monitored for weight loss and disease development (clinical score). Surprisingly, while both WT and *Cul5^fl/fl^* LysM-Cre mice started to show increased clinical scores by day 10 post-induction, disease in *Cul5^fl/fl^* LysM-Cre mice did not continue to progress (**Figure 2B**). Analysis at day 14 post-induction revealed that *Cul5^fl/fl^* LysM-Cre mice exhibited significantly lower clinical scores compared to their WT counterparts (**Figure 2B**). This time point corresponds to the peak of the disease in the EAE model. Consistent with this, while WT mice showed a reduction of weight as EAE developed, *Cul5^fl/fl^* LysM-Cre mice did not lose weight following EAE induction (**Figure 2C**).

Immune cell recruitment into the CNS is a key initiator of neuroinflammation, as it drives tissue damage and disease progression. To assess the types of cells in the CNS of WT and *Cul5^fl/fl^* LysM-Cre mice, we isolated cells from the CNS and analyzed the landscape of cell types present in the CNS using flow cytometry. As expected, WT mice showed an increase in CD4^+^ T cells over control mice, as expected following EAE induction (**Figure 2D**). In contrast, *Cul5^fl/fl^* LysM-Cre mice showed a significant reduction of CD4^+^ T cells in the CNS compared to EAE challenged WT mice (**Figure 2E**). *Cul5^fl/fl^* LysM-Cre mice also had decreased numbers of neutrophils (**Figure 2F**) and CD45^hi^ microglia/macrophages (**Figure 2G**), but not CD45^int^ microglia/macrophages (**Figure 2H**) when compared to WT mice. Based on published data, these CD45^int^ and CD45^hi^ cells are considered to be a broad representation of microglia/macrophages across reactive states. Furthermore, when we analyzed CD45^hi^MCHII^+^CD80^+^ microglia/macrophages, which represent activated macrophages with antigen-presenting characteristics, we again found fewer in *Cul5^fl/fl^* LysM-Cre mice compared to WT (**Figure 2I**). These findings suggest that *Cul5* deletion ameliorates immune cell accumulation in the CNS, thereby dampening inflammation and disease severity​.

### *Cul5* deletion in myeloid cells does not significantly impact activation or expansion of peripheral CD4^+^ T cells

CD4^+^ T cells are primary drivers of EAE pathogenesis. Peripheral activation of CD4^+^ T cells, and their differentiation into pro-inflammatory Th1 and Th17 subsets, occurs prior to their migration into the CNS, where they help to recruit neutrophils. Since a loss of Cul5 in macrophages might impact their ability to act as APCs, we next aimed to evaluate the peripheral activation of CD4^+^ T cells during the initiation of disease.

**Figure 3:**
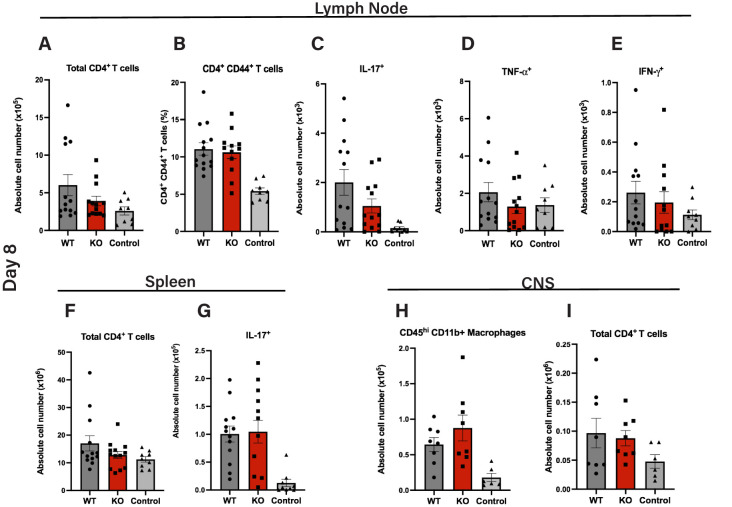
Similar induction of MOG_35–55_ peptide-specific T cells in peripheral lymphoid tissues in WT and Cul5^fl/fl^ LysM-Cre mice. Quantification of total numbers and MOG_35–55_-specific CD4^+^ T cell responses in cells isolated from secondary lymphoid tissues and CNS of *Cul5^fl/fl^* LysM-Cre and WT mice following *MOG_35–55_* immunization. Cells were isolated from the draining lymph nodes at day 8 post induction and analyzed by flow cytometry. Mean cell numbers of CD4^+^ T cells **(A)**, CD44+ CD4+ T cells **(B)**, and IL-17 **(C)**, TNFα **(D)**, and IFNγ **(E)** producing T cells. Cells were isolated from the spleen at day 8 post-induction and analyzed by flow cytometry, including total CD4+ T cells **(F)** and IL-17A+ CD4+ T cells **(G)** Cells were isolated from the CNS at day 8 post induction and analyzed by flow cytometry. Mean cell numbers of CD4^+^ T cells **(H)** and CD45^hi^ microglia/macrophages **(I)** in the CNS. Results are representative of three independent experiments **(A–F)** and two independent experiments **(G–H)** (n=10–11 mice per group). Error bars indicate mean ± SEM. Statistical significance was determined using an unpaired t-test (*P* < 0.05, P < 0.01, *P < 0.001, **P < 0.0001).

To assess whether *Cul5* deletion in myeloid cells influences CD4^+^ T cell activation and cytokine production, we analyzed cells in the draining lymph nodes (dLNs) and spleens as well as CNS on day 8 after induction of EAE. We isolated cells from the dLNs, spleens, and CNS from LysM-Cre and WT mice at day 8 post-immunization and quantified total numbers of CD4^+^ T cells as well as their cytokine-production. To assess cytokine production, we stimulated T cells with MOG_35–55_ *in vitro* and analyzed intracellular cytokine production by flow cytometry. While numbers of CD44+ ‘activated’ CD4+ T cells were significantly increased in both EAE cohorts over controls, we found that T cell numbers in the dLNs were comparable between *Cul5^fl/fl^* LysM-Cre and WT mice, as evidenced by similar absolute numbers of total CD4^+^ T and CD44+ ‘activated’ CD4^+^ T cells (**Figures 3A, B**). Furthermore, we found no significant differences in the numbers of MOG_35–55_ reactive IL-17^+^ (**Figure 3C**), TNF-α^+^ (**Figure 3D**), or IFN-γ^+^ (**Figure 3E**) producing T cells in dLNs between *Cul5^fl/fl^* LysM-Cre and WT mice. Furthermore, the overall numbers of T cells in the spleens were comparable between *Cul5^fl/fl^* LysM-Cre and WT mice (**Figure 3F**), as were the numbers of MOG_35–55_ reactive IL-17^+^ producing CD4^+^ T cells (**Figure 3G**).

Following peripheral activation, MOG reactive CD4^+^ T cells migrate into the CNS. Thus, we next sought to assess whether Cul5-deletion in myeloid cells impacted T cell migration into the CNS. On day 8 post EAE induction, cells from the CNS of WT and *Cul5^fl/fl^* LysM-Cre mice were analyzed using flow cytometry. We found that the number of cells in the CD45^hi^ gate was comparable between WT and *Cul5^fl/fl^* LysM-Cre mice (**Figure 3H**). Furthermore, the total number of CD4^+^ T cells was similar between WT and *Cul5^fl/fl^* LysM-Cre mice (**Figures 3I**). These data suggest that the loss of *Cul5* in myeloid cells had no significant impact in T cell priming or migration into the CNS following EAE induction. This is consistent with WT and *Cul5^fl/fl^* LysM-Cre mice showing a similar increase in disease score on days 9–11 post EAE induction.

### *Cul5* deletion leads to increased Arginase 1 production in macrophages during EAE

Once in the CNS, T cells accumulate in the perivascular space before fully infiltrating into the parenchyma (43). Local APCs, such as microglia and macrophages, present CNS-derived antigens to CD4^+^ T cells, thereby reactivating them (44). This local reactivation is essential for T cell persistence in the CNS and it enhances the release of pro-inflammatory cytokines, which further disrupts the BBB and recruits additional immune cells (45). Among these APCs, infiltrating macrophages play a crucial role by presenting CNS-derived antigens via MHCII and providing key costimulatory signals, such as CD80/CD86, to enhance T cell activation (46). This interaction is necessary to amplify the immune response, leading to further immune cell recruitment. Given that *Cul5^fl/fl^* LysM-Cre mice showed similar numbers of infiltrating T cells and macrophages on day 8 post EAE induction compared to WT mice, but showed decreased numbers of both types of cells by day 14 post-induction, we decided to further investigate macrophage numbers and phenotype on day 12, when *Cul5^fl/fl^* LysM-Cre mice are just starting to show a decrease in EAE disease severity.

**Figure 4:**
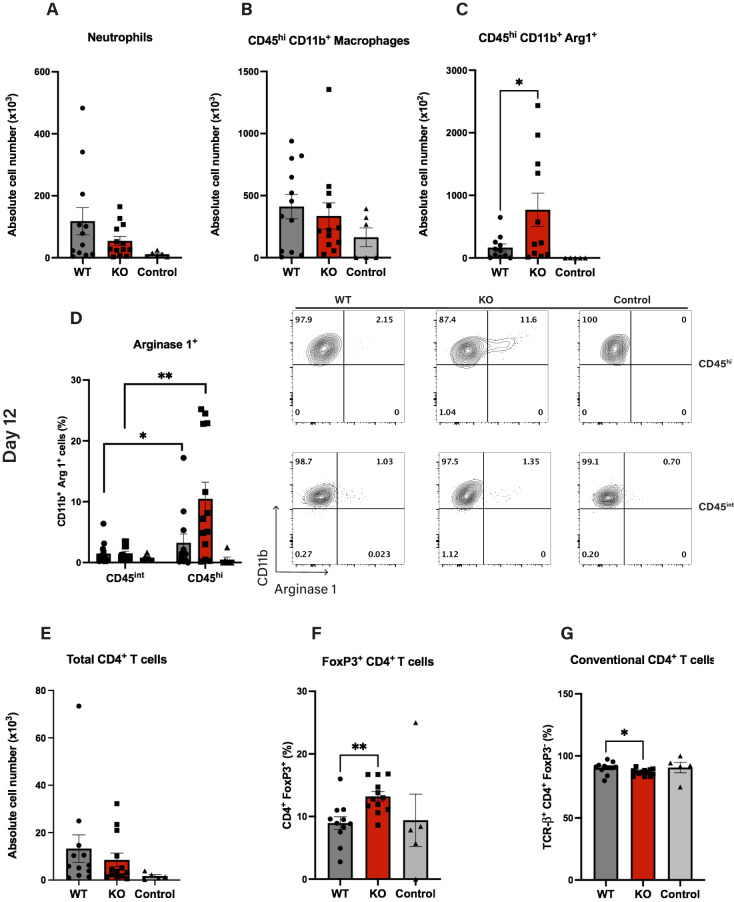
Cul5 deficiency enhances Arginase 1 expression in macrophages during EAE. Quantification of myeloid and lymphoid cell populations in the CNS of *Cul5fl/fl* LysM-Cre and WT mice at day 12 post-induction of EAE. **(A–C)** Total cell numbers of CD45^+^CD11b^+^ infiltrating myeloid cells, CD45^hi^ Arginase 1^+^ microglia/macrophages and Ly6G^+^neutrophils in the CNS following *MOG_35–55_* immunization. **(D, E)** Frequency of Arginase 1^+^ cells within the CD45^hi^ and CD45^int^ populations, highlighting the enrichment of Arginase 1-expressing in the CD45^hi^ subset in *Cul5*-deficient mice. **(F)** Mean total cell numbers of CD4^+^ T cells, **(F, G)** Frequency of conventional CD4^+^ T cells, and FoxP3^+^ regulatory T cells in the CNS following *MOG_35–55_* immunization. Data represent pooled results from two independent experiments (n=11 mice per group). Error bars indicate mean ± SEM. Statistical significance was determined using an unpaired t-test (*P* < 0.05, P < 0.01, *P < 0.001, **P < 0.0001. Each dot represents an individual mouse.

Specifically, WT and *Cul5^fl/fl^* LysM-Cre mice were induced with EAE and on day 12 cells were isolated from the CNS and analyzed by flow cytometry. We did not observe a significant difference in the overall numbers of CD45^hi^ microglia/macrophages (**Figure 4A**), or neutrophils (**Figure 4B**) in the CNS at this time point. However, when we analyzed the phenotype of CD45^hi^ microglia/macrophages, we found a significant increase in the number of Arginase 1 expressing cells in *Cul5^fl/fl^* LysM-Cre mice compared to WT (**Figure 4C**), suggesting that *Cul5*-deficient macrophages were biased toward an anti-inflammatory or reparative macrophage phenotype. Arginase 1 was predominantly expressed by CD45^hi^ cells, and not by CD45^int^ cells, consistent with a study by Greenhalgh et al. (47), where lineage tracing further identified CD45^hi^ cells as infiltrating monocytes. (**Figure 4D**). In our study, the increase in CD45^hi^/Arg1 cells correlated with a change in regulatory T cell frequencies. Specifically, while the overall number of CD4^+^ T cells was not different between *Cul5^fl/fl^* LysM-Cre mice compared to WT (**Figure 4E**), the frequency of FoxP3^+^ regulatory T cells (Tregs) was significantly higher in *Cul5^fl/fl^* LysM-Cre mice (**Figure 4F**) compared to WT controls. This increase in FoxP3^+^ Tregs, coupled with the decrease in conventional CD4^+^ T cells (**Figure 4G**) and enhanced presence of Arginase 1^+^ macrophages, supported that *Cul5* deficiency in myeloid cells may actively promote an anti-inflammatory state within the CNS.

### *Cul5* deficiency enhances Arginase 1 expression downstream of IL-4R signaling

**Figure 5:**
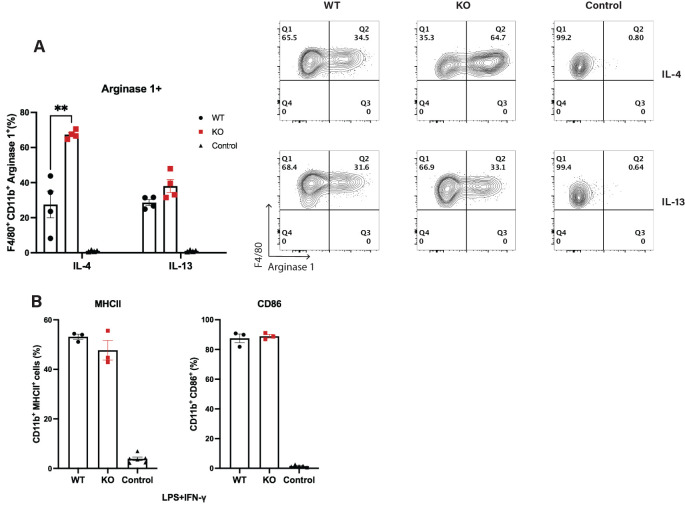
Loss of Cul5 results in increased Arginase 1 levels following IL-4 receptor stimulation of BMDMs. **(A)** BMDMs were generated from WT and *Cul5*-deficient mice. Cells were then analyzed for Arginase 1 levels via flow cytometry. Representative and compiled data showing the frequencies of Arginase 1^+^ cells following IL-4 or IL-13 stimulation (10 ng/mL) for 24 hours. **(B)** Percentage positive of CD86 and MHCII BMDMs following LPS (100 ng/mL) and IFN-γ (10 ng/mL) stimulation for 24 hours, showing no significant differences in upregulation of co-stimulatory molecules between *Cul5^fl/fl^* LysM-Cre and WT macrophages. Results are representative of three independent experiments (n=3–4 mice per group). Error bars indicate mean ± SEM. Statistical significance was determined using an unpaired t-test (**P < 0.01).

The production of Arginase 1 by myeloid subsets, particularly macrophages, creates a tissue environment with insufficient essential amino acid concentration which restricts T cell activation and proliferation (48, 49). Given the increase in Arginase 1 positive microglia/macrophages in *Cul5^fl/fl^* LysM-Cre mice, we next sought to test whether loss of Cul5 might bias macrophages toward an M2 phenotype *in vitro*. We examined Arginase 1 expression in WT and *Cul5*-deficient macrophages cultured in IL-4 or IL-13. BMDMs generated from WT and *Cul5^fl/fl^* LysM-Cre mice were cultured for 24 hours in either IL-4 or IL-13 and cells were analyzed by flow cytometry. Both IL-4 and IL-13 were able to drive expression of Arginase 1 in WT cells (**Figure 5A**). Interestingly, Cul5-deficient BMDMs showed a significant increase in the percentage of Arginase 1^+^ cells following IL-4 stimulation compared to WT cells (**Figure 5A**). In contrast, the percentages of Arginase 1^+^ macrophages were similar between the two genotypes when cells were cultured in IL-13 (**Figure 5A**). To determine whether CUL5 might also play a role in macrophage pro-inflammatory responses, we cultured WT and *Cul5^fl/fl^* LysM-Cre BMDMs with LPS and IFN-γ. Percentages of both CD80 and CD86 remained unchanged between groups, suggesting that Cul5 loss does not prevent a pro-inflammatory APC phenotype induction in macrophages (**Figure 5B**).

### Loss of *Cul5* triggers an anti-inflammatory protein expression profile in macrophages

**Figure 6:**
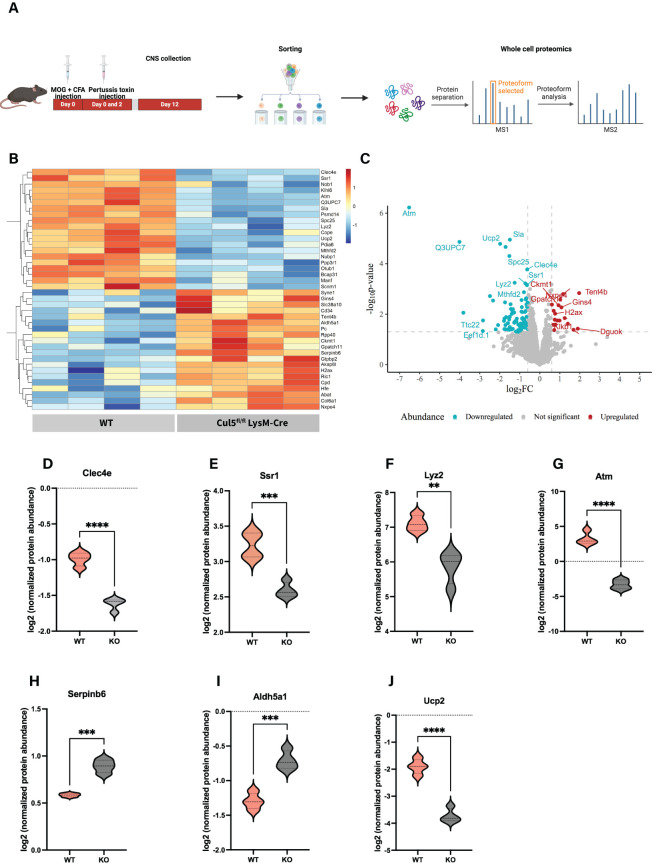
Loss of Cul5 triggers an anti-inflammatory protein expression profile in macrophages. **(A)** Workflow illustrating *ex vivo* cell isolation for WCP analysis **(B)** Heatmap displaying the top 20 significantly upregulated and downregulated proteins in *Cul5^fl/fl^* LysM-Cre vs WT CD45^hi^ microglia/macrophages. Each row represents a protein, and each column represents a biological replicate. Normalized Z score of protein abundance is depicted using a pseudocolor scale. **(C)** Volcano plot displaying the distribution of differentially expressed proteins between *Cul5^fl/fl^* LysM-Cre and WT macrophages. The x-axis represents the log2 fold change (log2FC) in protein abundance, while the y-axis represents the log10 (p-value), indicating statistical significance. Proteins significantly downregulated in *Cul5^fl/fl^* LysM-Cre are shown in blue, while those upregulated are shown in red. Proteins with no significant changes are represented in gray. Dashed horizontal lunes indicate significance and fold-change thresholds used for analysis. **(D-I)** show individual violin plots for **(D)** Clec4e, **(E)** Ssr1, **(F)** Lyz2, **(G)** Atm, **(H)** Serpinb6, **(I)** AIdh5a1 **(J)** Ucp2 displaying log2‐normalized protein abundances as measured by whole‐cell proteomics in WT and *Cul5^fl/fl^* LysM-Cre. Protein intensities were normalized and plotted for individual replicates. Violin plots represent the mean. Statistical analysis was performed using Student’s t-test (**P<0.01, ***P<0.001, ****P<0.0001).

To gain a deeper understanding of how Cul5 loss impacts CNS myeloid subsets, we conducted whole-cell proteomic analysis on WT and *Cul5*-deficient CD45^hi^ microglia/macrophages directly isolated from the CNS of EAE-induced mice at day 12 post-induction. To do this we employed high-resolution mass spectrometry (**Figure 6A**). Our CNS isolation and fluorescence-activated cell sorting (FACS) strategies yielded approximately 40,000 cells per sample, enabling the identification of an average of 4,000 proteins per condition (**Supplementary Figure S3**).

We applied a statistical threshold of p < 0.05 and a greater than 2-fold change to evaluate proteins significantly differentially expressed in *Cul5^fl/fl^* LysM-Cre macrophages compared to their WT counterparts (**Figures 6B, C**). Among the top 10 differentially expressed proteins, we observed a notable reduction in key mediators of pro-inflammatory responses, including *Clec4e* (**Figure 6D**), a C-type lectin receptor known to modulate macrophage activation and pathogen recognition (50), *Ssr1* (**Figure 6E**), a component of the translocon-associated protein complex that may contribute to antigen processing and MHC presentation (51); and *Lyz2*, a hallmark myeloid antimicrobial enzyme associated with phagocytosis and inflammatory responses (52, 53) (**Figure 6F**).

Interestingly, we also identified a significant downregulation of *Atm* (ataxia telangiectasia mutated kinase) in *Cul5*-deficient macrophages (**Figure 6G**). *Atm* plays an important role in DNA damage responses, cellular stress adaptation, response to metabolic changes and immune regulation (54–56). Moreover, ATM is crucial for shaping macrophage pro-inflammatory phenotype transition, by controlling IRF5 and its post-translational modifications (57). Together, these observations support the hypothesis that CUL5 has an important function in microglia/macrophages genomic stability and stress adaptation mechanisms in the CNS microenvironment.

Among the top upregulated proteins, we found *Serpinb6* (**Figure 6H**), which plays a role in cellular damage responses (58, 59). Additionally, *Cul5*-deficient cells showed upregulation of *Aldh5a1* (**Figure 6I**), a key enzyme in GABA metabolism and oxidative stress responses (4, 60), which may suggest a metabolic reprogramming or foamy macrophage-like features. These findings highlight that CUL5 plays an important role in the mechanisms by which CNS microglia/macrophage subsets function during neuroinflammation.

## Discussion

Macrophage phenotypic transition allows cells to rapidly respond to microenvironment signals and adapt accordingly. This plasticity is key during neuroinflammatory responses. Prior studies revealed that SOCS1 and SOCS3 play an anti-inflammatory role in macrophages and based on the association of SOCS proteins with CUL5, many researchers have assumed that CUL5 would play a similar role. Here we show an unanticipated pro-inflammatory role for *Cul5* in limiting the expansion of Arginase 1^+^ macrophages during neuroinflammation. Our results support that selectively deleting *Cul5* in myeloid cells alters macrophage phenotype and function, attenuating disease progression after inflammatory cells arrive in the CNS.

Our data show that *Cul5* deletion does not have a significant impact on myeloid cell development under homeostatic conditions (**Figures 1A–G**), contrasting with previously reported data on the roles of SOCS1 and SOCS3. Both are known regulators of JAK/STAT pathways, particularly downstream of GM-CSF, M-CSF, IL-6, which are important in myeloid development. This suggests that Cul5 and SOCS1/3 interactions might be dispensable for SOCS1/3-mediated regulation of myelopoiesis. However, surprisingly, C*ul5* deficiency in myeloid cells mitigated neuroinflammation, as evidenced by significantly lower clinical scores and reduced immune cell infiltration observed in *Cul5^fl/fl^* LysM-Cre mice. This included a decreased infiltration of CD4^+^ T cells and myeloid populations into the CNS during the peak of EAE (**Figures 2D–I**). This contrasts with previously characterized *Socs3^ΔLysM^* mice which exhibited an atypical and progressive EAE, characterized by infiltration of neutrophils and exacerbated neuroinflammation (33). These striking differences suggest that, rather than acting collaboratively with SOCS3, Cul5 and SOCS3 may have distinct or even opposing roles in regulating myeloid immune responses during neuroinflammation. While SOCS3 loss promotes an exacerbated inflammatory response, Cul5 deficiency appears to favor an immunosuppressive microenvironment.

Interestingly, despite the similar capacity for peripheral CD4^+^ T cell activation and cytokine production between *Cul5^fl/fl^* LysM-Cre and WT cohorts (**Figures 3A–E**), the immunosuppressive microenvironment in the CNS of *Cul5*-deficient mice, characterized by an increase in Arginase 1^+^ macrophages and FoxP3^+^ (**Figures 4C, F**) regulatory T cells, likely underlies the observed reduction in disease severity. Given that the CNS is an immune-privileged site with distinct metabolic, inflammatory, and cellular cues, it is possible that microglia/macrophages are exposed to specific signals—such as local cytokine gradients, metabolic constraints, or interactions with resident glial cells—that promote an immunosuppressive phenotype in a *Cul5*-deficient setting. It is well established that IL-4 signaling is one of the primary inducers of Arginase 1 expression in macrophages (61, 62). However, while Th1, Th17 and Th2 responses can coexist during EAE, the CNS microenvironment primarily exhibits a predominantly Th1 and Th17 landscape during the inflammatory phase (day 7–14 post-induction) (63), leading to low IL-4 levels until the resolution phase. This raises an important question about the unique aspects of the CNS microenvironment driving this distinct response specifically in CD45^hi^ cells. Understanding these CNS-specific factors may provide insight into the differential regulation of macrophage function across tissues and their role in resolving neuroinflammation.

The increased Arginase 1 levels in *Cul5*-deficient CD45^hi^ microglia/macrophages support the idea that *Cul5* may preferentially regulate the inflammatory-to-reparative transition in infiltrating myeloid subsets. Supporting that this is occurring in infiltrating myeloid cells rather than microglia, Greenhalgh et al. demonstrated that Arginase 1 is expressed exclusively by infiltrating myeloid cells but not resident microglia in models of spinal cord injury (SCI) and EAE (47). *Cul5* expression levels are high in both microglia and monocyte derived macrophages, this raising the possibility that Cul5 might function in both populations. Although we used a widely established categorization (64, 65) for our study as CD45^hi^ and CD45^int^ cells, distinguishing these populations without genetic fate mapping remains a major challenge and caveat in the field. Studies typically refer to microglia and infiltrating macrophages as CD45^int^ and CD45^hi^, respectively, but during neuroinflammation microglia can upregulate CD45. Signature genes such as Tmem119 and P2ry12 are helpful in identifying microglia under homeostatic conditions, but their expression dramatically reduces with inflammation, making them subject to similar limitations as CD45. Further studies using monocyte and microglia-specific targeting, such as Tmem119-CreERT2 and Ms4a3-Cre, are needed to fully discriminate infiltrating versus endogenous macrophages and elucidate how *Cul5* regulates cytokine signaling in microglia.

Arginase 1 helps to repair damaged tissues, as ornithine is a precursor to polyamines, which have pro-repair properties (66). Its activity depletes L-arginine from the microenvironment, thereby limiting the activity of pro-inflammatory Th1 and Th17 cells. This prominent feature not only limits inflammation but also fosters tissue repair and supports the expansion of regulatory FoxP3^+^ T cells (Tregs), consistent with the increase that we see in our data. The increase in FoxP3^+^ cells in *Cul5^fl/f^* ^l^LysM-Cre mice reinforces the notion that *Cul5* deficiency promotes an immunosuppressive environment within the CNS.

Our investigation into the receptor responsible for driving increased Arginase 1 production in *Cul5*-deficient macrophages supported that CUL5 might regulate IL-4 signaling. IL-4, but not IL-13, was sufficient to induce increased Arginase 1 levels in *Cul5*-deficient BMDMs (**Figure 5A**). These data suggested that CUL5 regulates the type I IL-4 receptor complex, distinct from the type II receptor used by IL-13. Both cytokines activate the JAK-STAT6 pathway, which is central to anti-inflammatory macrophage polarization. However, our findings suggest that CUL5-mediated ubiquitination may target specific components of the IL-4 signaling cascade, thereby modulating macrophage function, as was seen previously with CD4+ T cells (39).

Consistent with the increase in Arginase 1, we also identified a set of anti-inflammatory features associated with *Cul5*-deficient macrophages isolated from the CNS. Our whole-cell proteome data (**Figures 6B, C**) revealed a distinct shift in *Cul5^fl/f^* ^l^ LysM-Cre macrophage phenotypic characteristics toward an anti-inflammatory state. Key inflammatory genes, such as *Clec4e* and *Lyz2* were significantly downregulated in *Cul5*-deficient macrophages, pointing to a reduction in pro-inflammatory associated immune activation. However, a limitation in our strategy is the reliance on CD45^hi^ and CD45^int^ gating to define these populations, since it does not fully exclude other subsets such as activated microglia and monocyte-derived dendritic cells. As such, our proteomics data may reflect a heterogenous population. Further studies using more specific sorting strategies and single-cell resolution will be important to fully dissect the functional contributions of these myeloid populations.

Together, these findings might explain why *Cul5*-deficient macrophages improved disease outcomes in EAE. Interestingly, the role we describe here for CUL5 in macrophage-mediated neuroinflammation has similarities to work that was recently reported in anti-viral immune responses (67). Specifically, following viral infection, *Cul5* deficient macrophages prevented neutrophilic accumulation in the lung and protected against viral-mediated asthma exacerbation (67). Together, these data support that CUL5 promotes pro-inflammatory macrophage function following viral infection, and during autoimmune disease development.

A final limitation of this study is the assessment of CUL5 expression in macrophages from MS patient samples. Although our findings in the EAE model suggest an important role for CUL5 in modulating microglia/macrophage phenotype and function, further studies are needed to determine whether similar regulatory mechanisms are conserved in human MS. Publicly datasets may offer insights, but they often lack cell-type resolution. In conclusion, CUL5 emerges as a key regulator of macrophage-mediated immune responses and thus impacts neuroinflammation. To date, no MS therapies specifically target macrophages as a therapeutic strategy. Additionally, the specificity of CUL5 regulation of IL-4-induced Arginase 1 expression provides an opportunity to fine-tune therapeutic strategies to modulate macrophage phenotypes without broadly suppressing overall immune responses. The findings presented here represent an important first step in understanding the role of CUL5 in neuroinflammation and establishing a foundation for future research into its therapeutic potential in MS and other autoimmune diseases.

## Data Availability

The mass spectrometry proteomics data have been deposited to the ProteomeXchange Consortium via the PRIDE partner repository with the dataset identifier PXD066533.

## Supplementary material

Supplementary Figure 1 Cul5 does not broadly affect the differentiation of myeloid cells under homeostatic conditions. **(A–C)** Immunophenotyping of myeloid and lymphoid cell populations in the spleen, bone marrow, and brain of *Cul5^fl/fl^* LysM-Cre and WT mice under homeostatic conditions. Bars indicate mean ± SEM. Data were analyzed using multiple unpaired t-tests and are representative of two independent experiments. Each dot represents an individual mouse.

Supplementary Figure 2 Cul5 expression impacts CD4^+^ T cell frequency in the CNS and spleen during EAE. Quantification of immune cell populations in the CNS and spleen of *Cul5^fl/fl^* LysM-Cre and WT mice at day 14 post-induction of EAE. **(A–D)** Frequencies of CD4^+^ T cells, CD45^hi^ and CD45^int^ cells and neutrophils in the CNS. **(E–H)** Frequencies of TCR-β^+^ T cells, CD19^+^ B cells, neutrophils, and CD4^+^ T cells in the spleen. Data are representative of two independent experiments. Bars indicate mean ± SEM. Statistical significance was determined using an unpaired t-test (*P* < 0.05, P < 0.01, *P < 0.001, **P < 0.0001).

Supplementary Figure 3 Comparison of protein identification across WT and Cul5^fl/fl^ LysM-Cre CD45^hi^ microglia/macrophages. **(A)** Bar plot displaying the total number of identified proteins per sample in WT (blue) and *Cul5^fl/fl^* LysM-Cre (green) conditions. Each bar represents an individual biological replicate.
