# Supplementary figures and images for: Loss of Cullin 5 in myeloid cells protects against autoimmune neuroinflammation
Source: Front Immunol. 2025 Aug 6;16:1611818. doi: 10.3389/fimmu.2025.1611818 (PMC12366467; doi:10.3389/fimmu.2025.1611818)

S. Figure 1

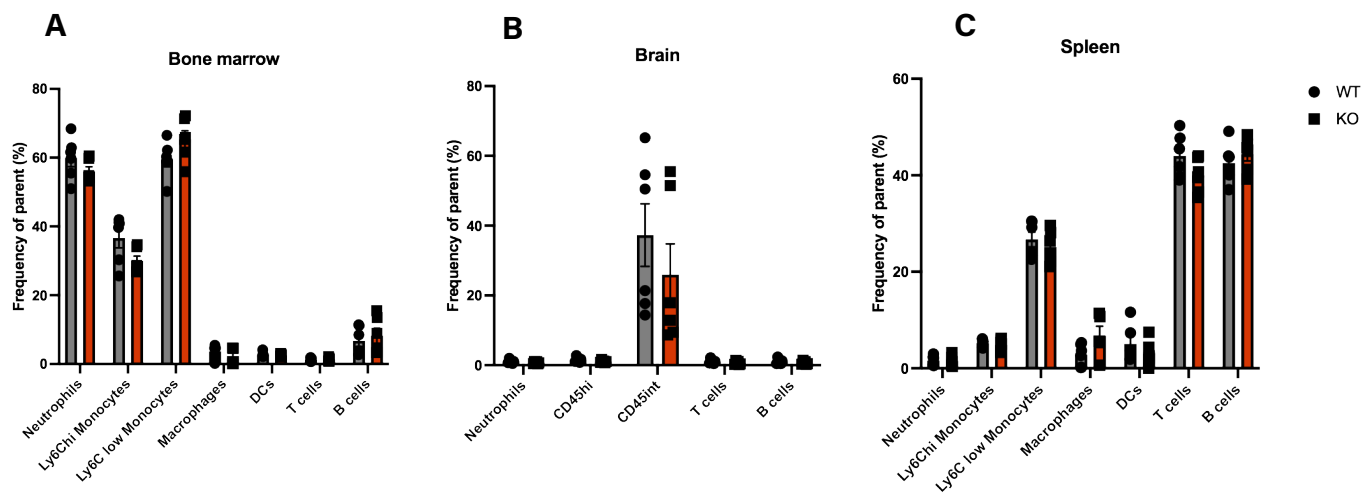

Supplement: Supplementary Figure 1 — Cul5 does not broadly affect the differentiation of myeloid cells under homeostatic conditions. (A–C) Immunophenotyping of myeloid and lymphoid cell populations in the spleen, bone marrow, and brain of Cul5fl/fl LysM-Cre and WT mice under homeostatic conditions. Bars indicate mean ± SEM. Data were analyzed using multiple unpaired t-tests and are representative of two independent experiments. Each dot represents an individual mouse. [file Image1.pdf]

S. Figure 2

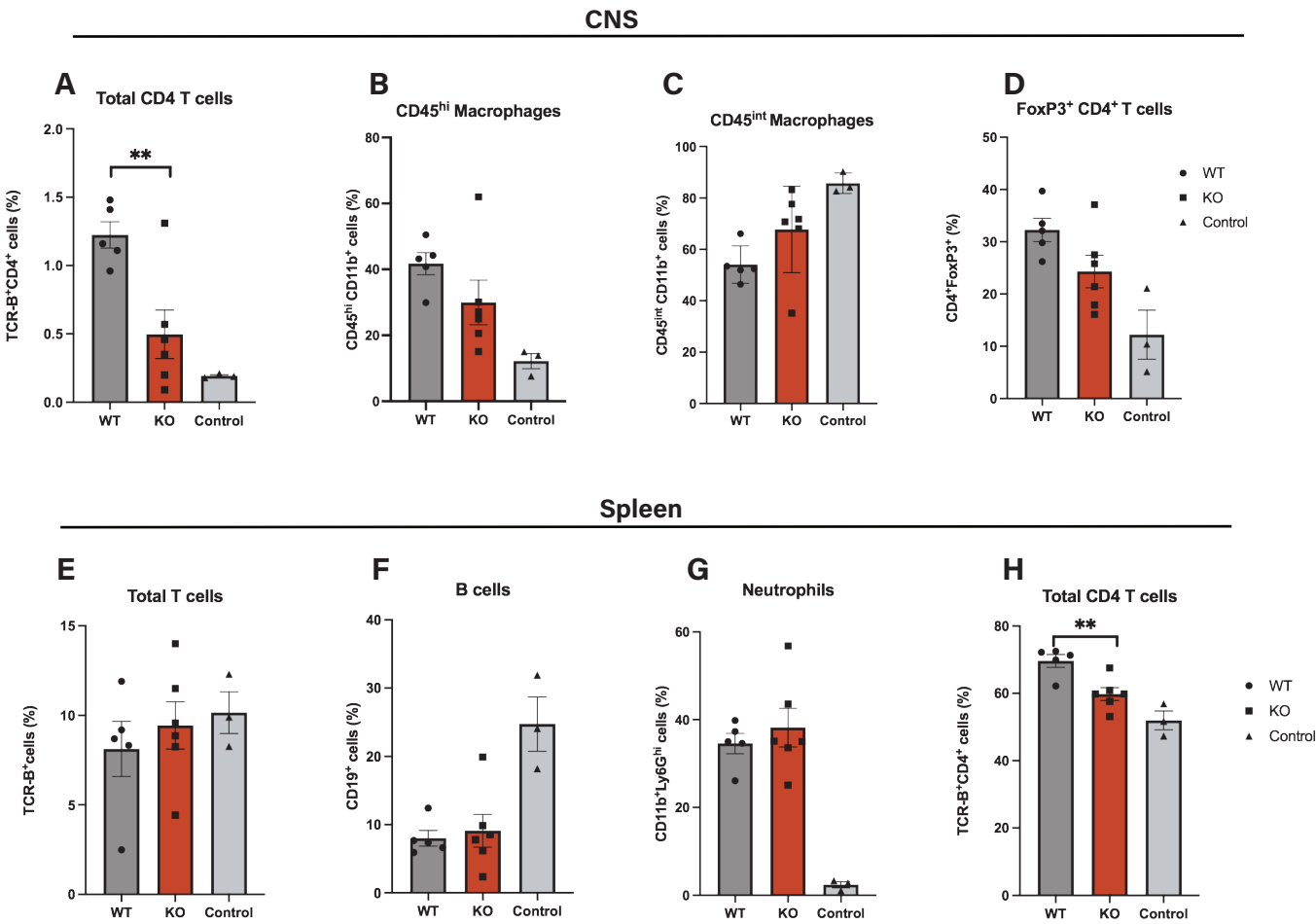

Supplement: Supplementary Figure 2 — Cul5 expression impacts CD4+ T cell frequency in the CNS and spleen during EAE. Quantification of immune cell populations in the CNS and spleen of Cul5fl/fl LysM-Cre and WT mice at day 14 post-induction of EAE. (A–D) Frequencies of CD4+ T cells, CD45hi and CD45int cells and neutrophils in the CNS. (E–H) Frequencies of TCR-β+ T cells, CD19+ B cells, neutrophils, and CD4+ T cells in the spleen. Data are representative of two independent experiments. Bars indicate mean ± SEM. Statistical significance was determined using an unpaired t-test (P < 0.05, P < 0.01, *P < 0.001, **P < 0.0001). [file Image2.pdf]

S. Figure 3

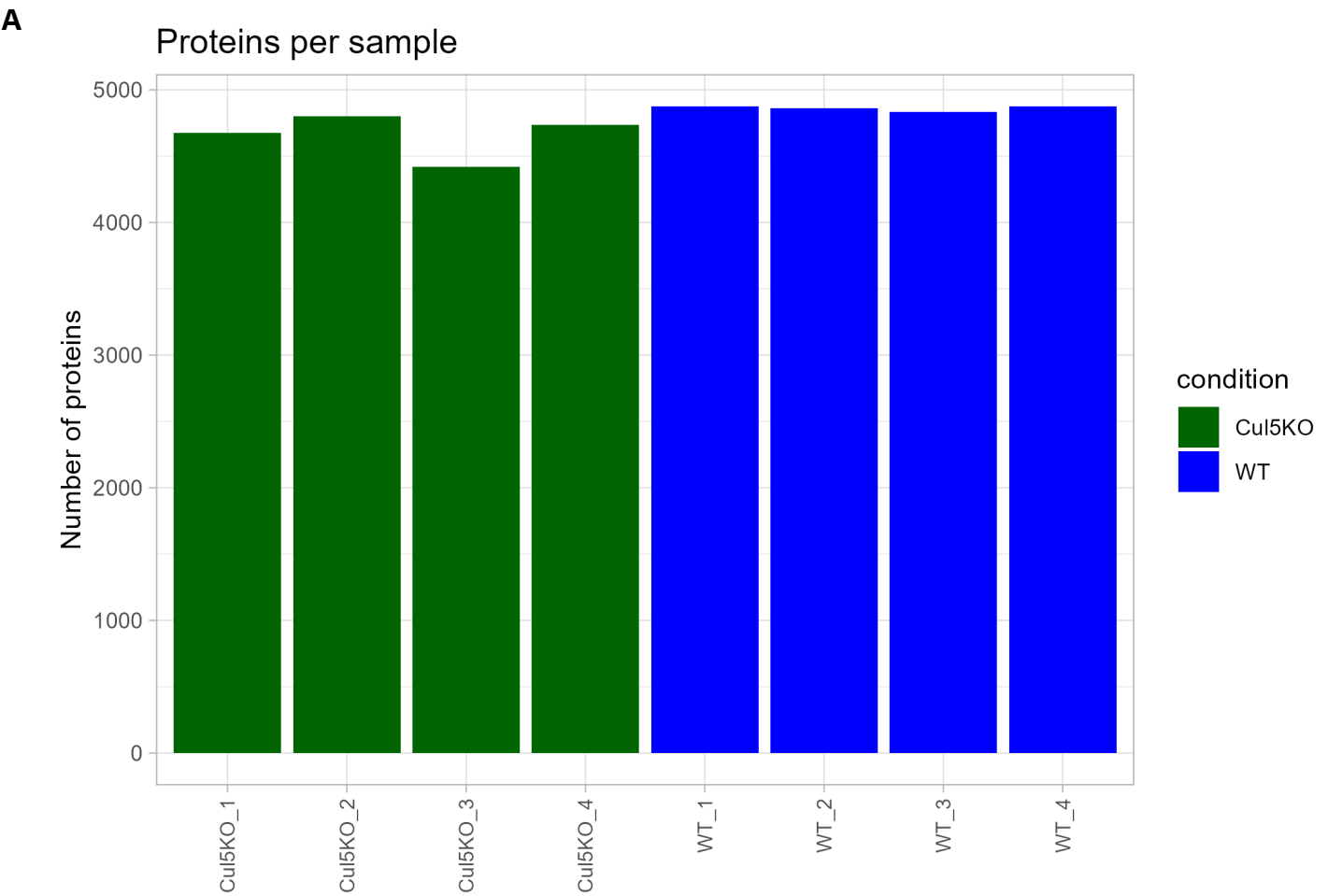

Supplement: Supplementary Figure 3 — Comparison of protein identification across WT and Cul5fl/fl LysM-Cre CD45hi microglia/macrophages. (A) Bar plot displaying the total number of identified proteins per sample in WT (blue) and Cul5fl/fl LysM-Cre (green) conditions. Each bar represents an individual biological replicate. [file Image3.pdf]
