# Supplementary material for: Loss of Cullin 5 in myeloid cells protects against autoimmune neuroinflammation
Source: Front Immunol. 2025 Aug 6;16:1611818. doi: 10.3389/fimmu.2025.1611818 (PMC12366467; doi:10.3389/fimmu.2025.1611818)

S. Figure 4

Figure 1: Bone marrow-derived macrophages

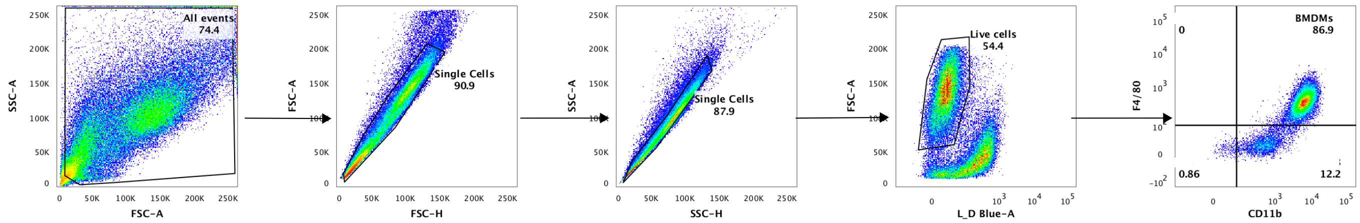

Figure 1 & S. Figure 1: Myeloid and lymphoid populations

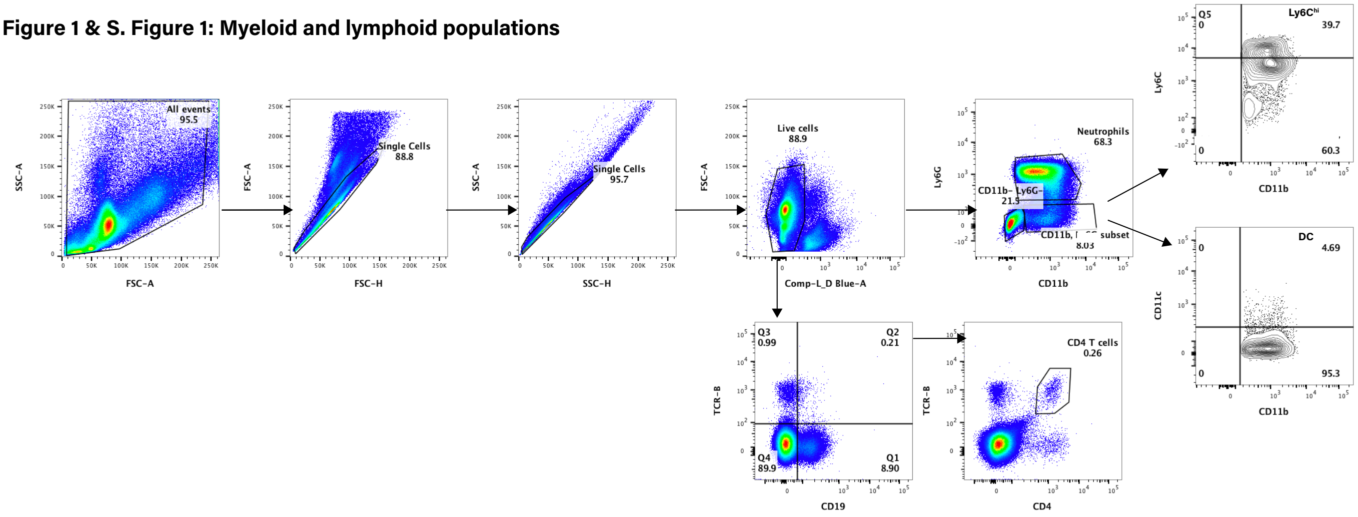

Figure 2: Myeloid and lymphoid populations

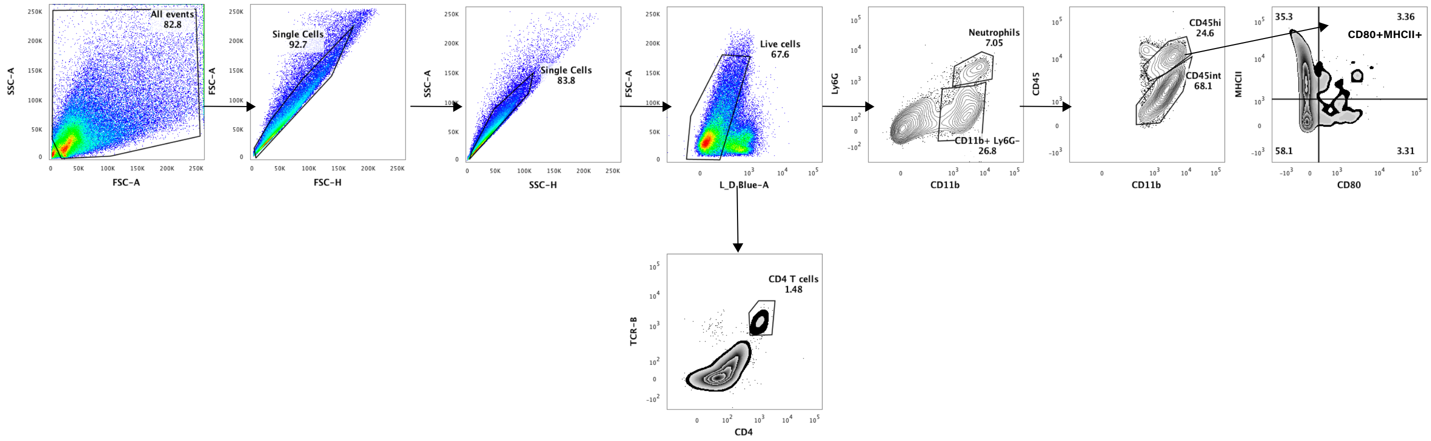

Figure 3: Myeloid and lymphoid populations

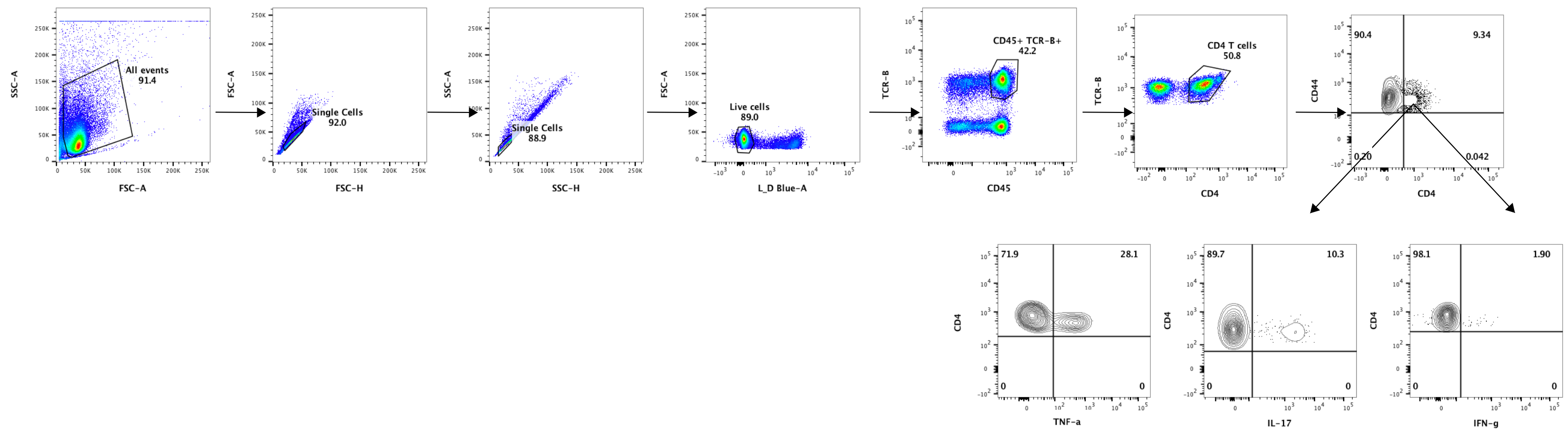

S. Figure 4

Figure 4: Myeloid and lymphoid populations

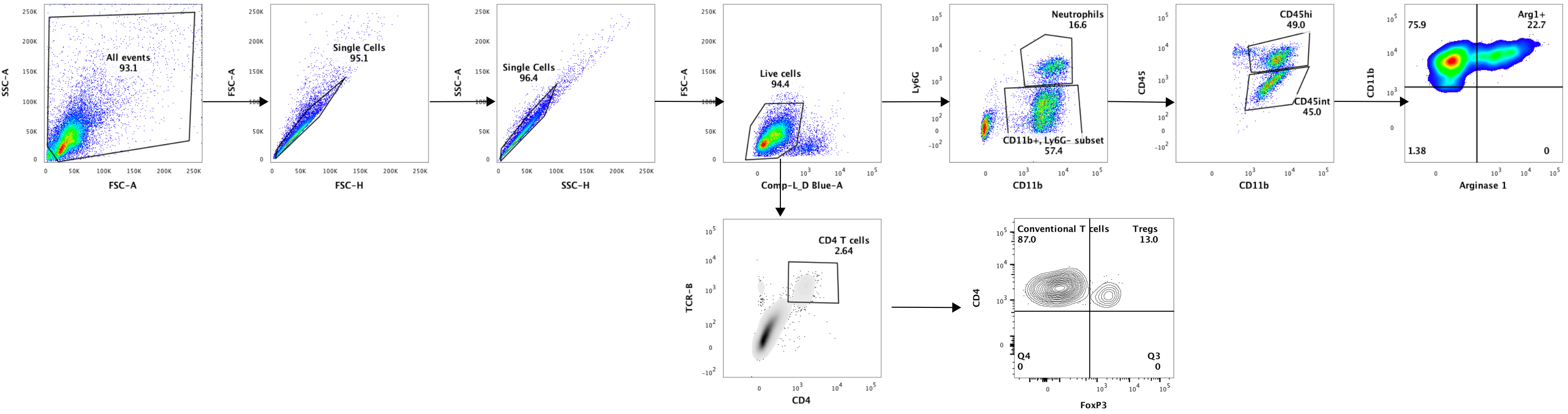

Figure 5: Bone marrow-derived macrophages

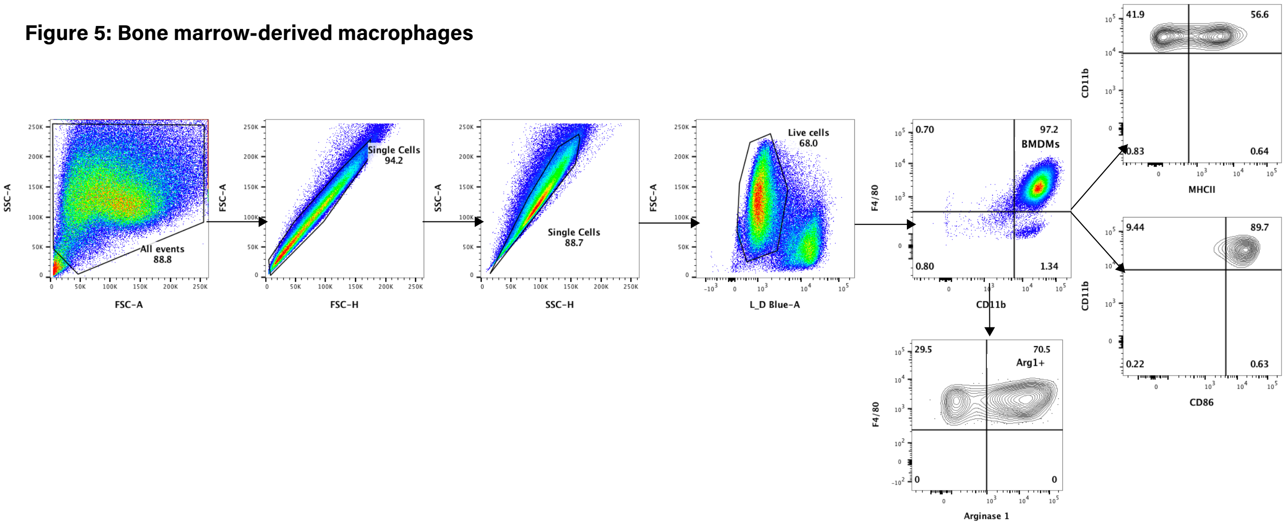

Supplement: Supplementary file 4 [file Image4.pdf]
